# Metastasis in the mandibular condyle: a case report

**DOI:** 10.1186/s13256-017-1450-9

**Published:** 2017-11-16

**Authors:** Mina Dodo, Masahiro Kumagai, Yuta Kato, Hisashi Hirakawa, Takeyoshi Koseki

**Affiliations:** 1 Division of Preventive Dentistry, Department of Oral Health and Development Sciences, Tohoku University Graduate School of Dentistry, Sendai, Japan; 2 Department of Oral and Maxillofacial Surgery, Tohoku Kosai Hospital, Sendai, Japan; 3 Department of Dentistry and Oral and Maxillofacial Surgery, Hamamatsu University School of Medicine, Hamamatsu, Japan; 4 Department of Surgery, Tohoku Kosai Hospital, Sendai, Japan

**Keywords:** Temporomandibular joint, Metastasis, Radiographic finding

## Abstract

**Background:**

Most bone metastases are observed in the trunk of the body. Metastasis in the mandibular condyle is rare. In many case reports, temporary common temporomandibular joint disorder-like symptoms can be a sign of relapse and metastasis.

**Case presentation:**

We report a rare case of breast carcinoma metastatic to the left mandibular condyle in a 55-year-old Japanese woman, who visited our department for a dental check-up prior to chemotherapy. She had almost no symptoms, but radiographs suggested the existence of metastasis.

**Conclusions:**

In many case reports, patients had some symptoms. In this case report, our patient had slight symptoms, but we were able to confirm the metastasis from the symptoms and panoramic dental radiograph. When patients complain about discomfort of the temporomandibular joint, we need to consider the possibility of metastasis and notice changes on the panoramic dental radiograph.

## Background

Bone metastasis is reportedly found in 65 to 75% of patients with advanced-stage breast cancer. Most bone metastases are observed in the trunk of the body. In contrast, metastasis in the mandibular condyle is rare. The purpose of this study is to present a case of a patient with metastasis in the left mandibular condyle originating from breast cancer that showed slight and temporary common temporomandibular joint disorder (TMD)-like symptoms.

Case reports of metastatic lesions in the mandibular condyle that were written in English and published between 2000 and 2016 were identified through searches of databases. The clinical and radiographic characteristics of the metastatic lesions in the mandibular condyle were discussed.

## Case presentation

A 55-year-old Japanese woman visited our department for a dental check-up prior to chemotherapy for breast cancer. She had a medical history of ovarian tumor, and her ovary and uterus had been removed. One month before (August 2009), at the same time that she felt stiffness in her left breast, she felt sudden trismus and difficulty with chewing. However, she had no pain and gradually recovered from these symptoms without any treatment.

At the initial consultation (September 30, 2009), she had no trismus, pain, or swelling, but showed a slight deviation to the left side when she opened her mouth. The maximum incisal opening distance was 40mm. A tumor-forming ulcer (10cm × 9cm) was observed in her breast. Positron emission tomography (PET) showed the tumor with a maximum standardized uptake value (SUV_max_) of 18 filling most of her right breast. Metastatic lymph nodes, bone lesions, and a lung lesion were also observed.

**Fig. 1:**
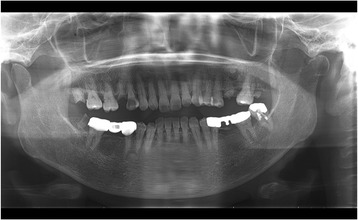
Panoramic dental radiograph. A radiolucent destructive lesion and pathologic fracture were seen in the left condyle

**Fig. 2:**
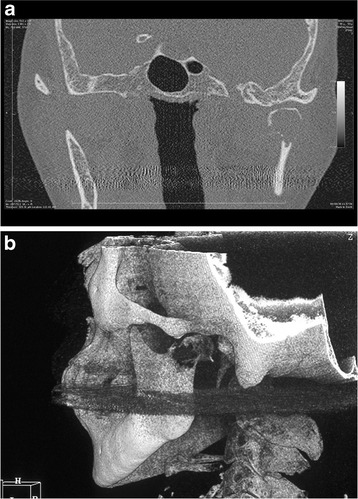
Computed tomography radiographs. **a** Computed tomography radiograph (coronal plane). A radiolucent destructive lesion of the left condyle was observed. The cortical bone at the condyle was thin and partially fractured. **b** Computed tomography radiograph (three-dimensional reconstruction). Destruction of the left mandibular condyle was observed

**Fig. 3:**
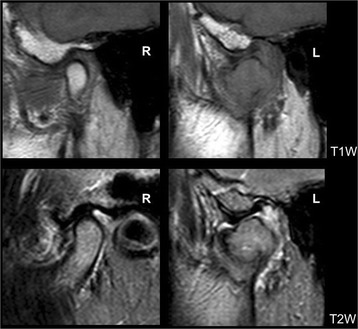
Magnetic resonance image. The upper images were T1-weighted and the lower images were T2-weighted. On the right side, the shape of the condyle was normal. An unstructured mass was observed in the left condylar region. A normal-shaped articular disc was seen on the destructed condyle

**Fig. 4:**
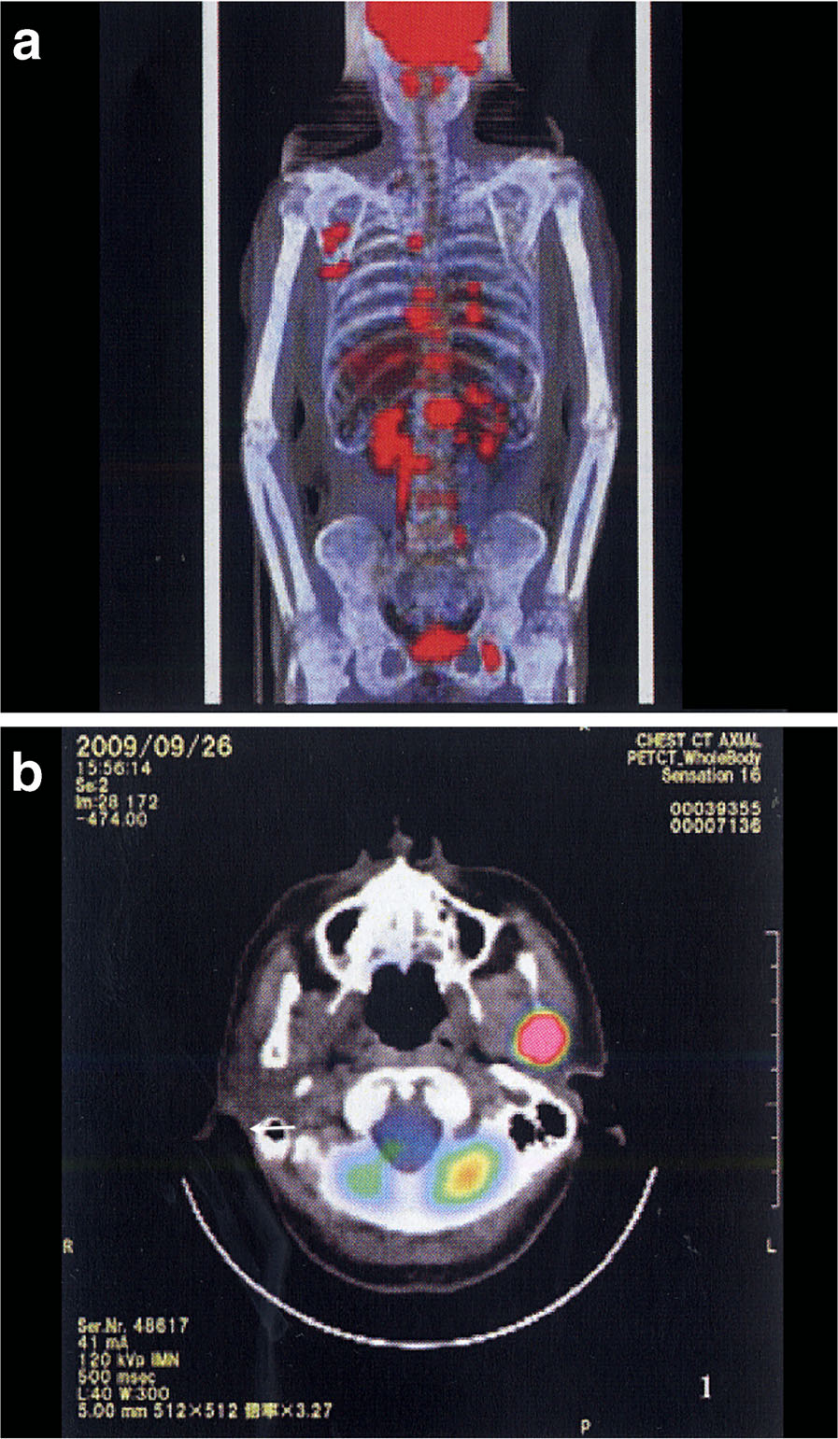
Positron emission tomography. Positron emission tomography showed multiple high-uptake lesions, including one in the left temporomandibular joint condyle

A panoramic dental radiograph and computed tomography (CT) radiograph showed the destruction of the left mandibular condyle (Figs. 1 and 2). On magnetic resonance imaging (MRI), a relatively well-defined unstructured mass was observed on both T1-weighted (T1W) and T2-weighted (T2W) imaging (Fig. 3). PET showed multiple lesions, including in the vertebrae, costal bones, and left mandibular condyle (Fig. 4). Given the diagnosis of left breast cancer associated with multiple metastatic lesions, chemotherapy using fluorouracil, epirubicin and cyclophosphamide (FEC treatment) was employed. In 4 months, when six cycles of chemotherapy had been completed, a radiograph showed a reduction in the size of both the primary and the metastatic lesions, including the left mandibular condylar lesion. An additional two cycles of chemotherapy were performed. Her general condition deteriorated. One and a half years after the first medical examination, she died.

## Discussion

As PET showed multiple bone masses in addition to the extended primary left breast lesion, our patient was diagnosed with breast cancer associated with multiple metastases. A biopsy of a sample from the left condyle was not performed, and pathologic diagnosis of the left condylar lesion was not available. Nevertheless, after the FEC treatment, the lesions, including the one in the left condyle, appeared to be reduced on the radiograph investigation, which suggested that the left condylar lesion as well as the multiple bone lesions had the same characteristics as the breast adenocarcinoma. From this clinical finding, the left condylar lesion was speculated to have been a metastatic lesion from the breast cancer.

Between 2000 and 2016, 19 case reports (24 cases) of metastasis to the condyle were published. The primary tumor was diagnosed as breast cancer in five cases, lung cancer in five cases, and prostate, colon, and kidney cancer in two cases, respectively. According to the reports (Table 1), at the first consultation, most of the cases involved obvious subjective symptoms such as pain (16 out of 22), swelling (15 out of 22), and trismus (8 out of 22). In most of the cases (six out of seven), a panoramic dental radiograph was performed, and bone destruction and absorption were recognized. In this case, our patient visited our department for a dental check-up before receiving chemotherapy for breast cancer. She did not have any severe symptoms at the first consultation. However, the condylar lesion was recognized at the same time as multiple other metastases on PET. A panoramic dental radiograph showed destruction. Several previous reports (10 out of 22) claimed that the mandibular condyle could be the first recognized metastatic region. Additionally, other reports (12 out of 22) stated that TMD-like symptoms can be a sign of relapse and metastasis. Because of the development of cancer treatment, the number of patients with a history of cancer treatment is increasing. When patients complain about discomfort of the temporomandibular joint, we need to consider the possibility of metastasis and notice changes on the panoramic dental radiograph.

**Table 1:** Case reports of metastatic tumors of the temporomandibular joint (from 2000 to 2013)

Primary lesion	Sex	Age (years)	Pain	Swelling	Trismus	Other complaints	^*^1	^*^2	Author
Bladder	M	49	○	○			☆		[1]
Prostate	M	85		○		Discomfort	☆		
Lung	F	62	○			Lower lip numbness	☆		
Penis	M	53		○		Numbness		☆	
Colon	M	64	○	○				☆	
Breast	F	47	○					☆	
Renal cell	F	59		○	○			☆	[2]
Breast	M	73	○	○			☆		[3]
Prostate	M	75	○	○	○			☆	[4]
Lung	M	49	○	○			☆		[5]
Breast	F	51	○	○	○			☆	[6]
Lung	M	51	○	○	○		☆		[7]
Breast	F	42	○	○				☆	[8]
Lung	M	60			○	Limitation of mandibular movement	☆		[9]
Breast	F	78	○		○			☆	[10]
Stomach	M	67				Progressive facial asymmetry		☆	[11]
Cystosarcoma	F	58	○			Hearing difficulty		☆	[12]
Lung	F	71	○			Changes in occlusion and functional limitation	☆		[13]
Chordoma	F	63	○	○	○			☆	[14]
Renal cell	M	49				An enlarging painless left mandibular mass	☆		[15]
Colon	M	73	○	○			☆		[16]
Liver	M	59	○	○	○			☆	[17]
Uterine cervix	F	65	○	○	○			☆	[18]
Uterine cervix	F	63	○	○				☆	[19]

^*^1:☆=the cases whose temporomandibular joint lesion was found before the primary lesion was recognized

^*^2:☆=the cases whose temporomandibular joint symptoms was a sign of a relapse and metastasis of the disease

*M* male, *F* female, *○* subjective symptoms

## Conclusions

We report a case of a patient with metastatic lesion in the mandibular condyle from breast cancer. Our patient had slight and common TMD-like symptoms. Dentists and oral surgeons should keep in mind the possibility of metastatic lesion in the mandibular condyle and should be familiar with its clinical and radiographic characteristics.
